# Polysaccharide-capped silver Nanoparticles inhibit biofilm formation and eliminate multi-drug-resistant bacteria by disrupting bacterial cytoskeleton with reduced cytotoxicity towards mammalian cells

**DOI:** 10.1038/srep24929

**Published:** 2016-04-29

**Authors:** Sridhar Sanyasi, Rakesh Kumar Majhi, Satish Kumar, Mitali Mishra, Arnab Ghosh, Mrutyunjay Suar, Parlapalli Venkata Satyam, Harapriya Mohapatra, Chandan Goswami, Luna Goswami

**Affiliations:** 1 School of Biotechnology, KIIT University, Patia, Bhubaneswar 751024, India; 2 School of Biological Sciences, National Institute of Science Education and Research, Institute of Physics Campus, Sachivalaya Marg, Bhubaneswar 751005, India; 3 Institute of Physics, Sachivalaya Marg, Bhubaneswar 751005, India

## Abstract

Development of effective anti-microbial therapeutics has been hindered by the emergence of bacterial strains with multi-drug resistance and biofilm formation capabilities. In this article, we report an efficient green synthesis of silver nanoparticle (AgNP) by *in situ* reduction and capping with a semi-synthetic polysaccharide-based biopolymer (carboxymethyl tamarind polysaccharide). The CMT-capped AgNPs were characterized by UV, DLS, FE-SEM, EDX and HR-TEM. These AgNPs have average particle size of ~20–40 nm, and show long time stability, indicated by their unchanged SPR and Zeta-potential values. These AgNPs inhibit growth and biofilm formation of both Gram positive (*B. subtilis*) and Gram negative (*E. coli* and *Salmonella typhimurium*) bacterial strains even at concentrations much lower than the minimum inhibitory concentration (MIC) breakpoints of antibiotics, but show reduced or no cytotoxicity against mammalian cells. These AgNPs alter expression and positioning of bacterial cytoskeletal proteins FtsZ and FtsA. CMT-capped AgNPs can effectively block growth of several clinical isolates and MDR strains representing different genera and resistant towards multiple antibiotics belonging to different classes. We propose that the CMT-capped AgNPs can have potential bio-medical application against multi-drug-resistant microbes with minimal cytotoxicity towards mammalian cells.

Infections caused by pathogenic bacteria have become a serious health and economic problem^1^. There has been constant decrease in effectiveness of antibiotics mainly due to unregulated use of antibiotics, leading to the development of multi-drug-resistant (MDR) bacterial strains^2^,^3^. Therefore, it has become necessary to search for alternative healthcare approaches to mitigate the problem of bacterial infections and contaminations. Typically, the bacterial infections can be categorised into two types, namely acute infection and chronic infections. The former, however are treated effectively by the development of modern vaccines, antibiotics and infection control measures^4^. However, the other type of infections has accentuated the infection related complications and therefore has poised a major challenge in controlling infection related issues^4^. Moreover, the treatment of acute infections have become difficult because the infection related diseases have been supplemented by chronic infections caused by bacteria growing in slime-enclosed aggregates known as biofilms^1^. Microbial infections which gets complicated due to biofilm formation, such as pneumonia in cystic fibrosis patients, chronic wounds, chronic otitis media and implant/catheter-associated infections, affect millions of people leading to death. While all existing approved antibiotics lose their efficacy against different bacterial strains rapidly, different nanoparticles with improved properties have been proposed as potential alternatives due to their broad range of antimicrobial activities^5^,^6^.

Nanoparticles exhibit completely new or improved properties based on specific characteristics such as size, distribution, morphology and exact chemical composition^7^. Certain unique properties such as optoelectronic and physicochemical properties of different NPs have already been successfully exploited in biomedical fields, especially for the purpose of drug delivery, tissue/tumour imaging, catalysis, bio-sensing and even for the development of different surface-enhanced Raman scattering-based sensors^8^,^9^,^10^. In this context, colloidal nanoparticles (NPs) have also attracted attention due to their ever-emerging, numerous, and fascinating applications in various fields of biomedical applications, especially as suitable antimicrobial agents^11^,^12^,^13^,^14^. In-spite of broad-range activities, successful and potential biomedical applications of AgNPs are hindered by several factors such as low stability of bare AgNPs and extremely high-level cytotoxicity. The molecular mechanism by which AgNPs in general inhibit different microbial growth is not well understood^1^,^15^,^16^. However, the effect of different AgNPs on eukaryotes and prokaryotes are somehow different and depends on the dose as well as the nature and properties of the target cell^15^,^16^,^17^. Therefore successful application of different AgNPs against bacterial infection in animal and/or in human not only needs development of improved and next generation AgNPs with better properties but also enforces the need for detailed understanding of the molecular mechanism by which AgNPs interact with different host cells as well as pathogens.

In this work we have taken a unique “green-synthesis” approach and prepared Carboxy Methyl Tamarind Polysaccharide-capped AgNPs which have better properties than the currently available AgNPs. We also explored the molecular mechanisms by which these AgNPs execute antimicrobial activities.

## Results

### Synthesis of CMT-capped AgNPs

Recent research on green synthesis of nanomaterials using different biopolymers has drawn considerable interest owing to its several applications which are better than chemically synthesized NPs^18^. In this present study we have synthesized silver nanoparticles (AgNPs) using aqueous solution of carboxy methyl tamarind polysaccharide as a reductant and capping agent. The optimization of AgNP synthesis was achieved by varying the concentration of CMT polysaccharide solution and silver nitrate solution alternatively (Supplementary Table 1).

**Figure 1:**
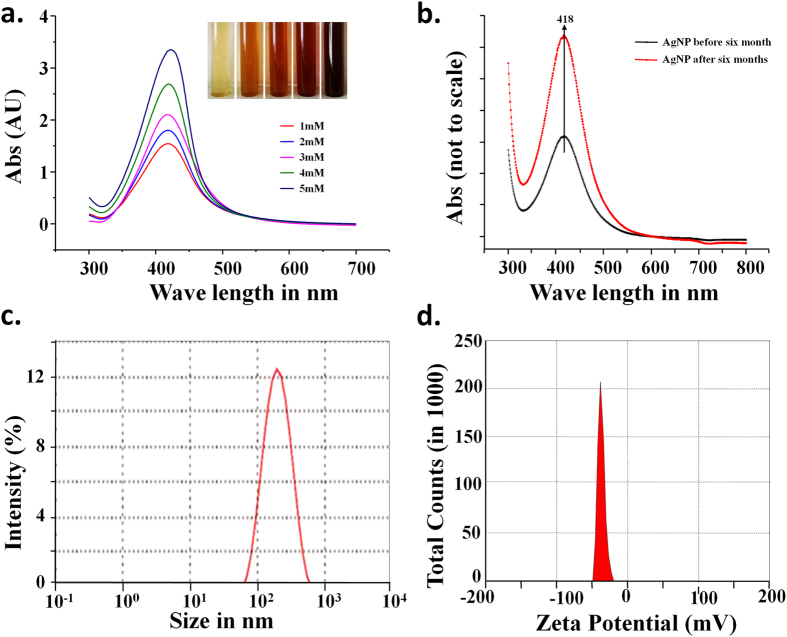
Synthesis and characterization of CMT-capped AgNPs. (**a**) UV-Visible spectra of silver with concentration of silver nitrate (1 to 5 mM) shows increase in intensity with increasing concentration of silver nitrate. A photo graph of test tubes containing silver nanoparticle synthesized from different concentration of AgNO_3_ (1 to 5 mM) with a fixed concentration of CMT polysaccharide is shown inset. (**b**) UV-Visible spectra showing unchanged SPR for silver nanoparticles before and after six months of synthesis of AgNPs. (**c**) Size distribution of silver NP as studied by DLS. (**d**) Zeta potential as measured by DLS showing a value of −36 mV which is well within the range for higher stability.

Figure 1a demonstrates the formation of AgNPs by *in situ* reduction of silver nitrate in presence of CMT polysaccharide. The colour changes observed in the aqueous solution may be attributed to the surface plasmon resonance (SPR) of synthesized AgNPs. As depicted in the figure, the colour gradually changes from yellow to brown and then to dark brown indicating the qualitative and quantitative changes in the AgNPs formed^19^. Formation of the darker coloured solution correlates well with increase in Silver Nitrate concentrations and this may be due to better conversion (better nucleation) of AgNPs from silver ion. There is no colour change observed for only CMT polysaccharide solution under similar conditions.

### Spectroscopic characterization of CMT-capped AgNPs

The UV-visible spectroscopy is widely used as a useful technique for studying the nanoparticles owing to the characteristic surface plasmon resonance observed for different metal nanoparticles including AgNPs. Figure 1a shows the UV-visible absorbance spectrum for synthesized CMT-capped AgNPs having surface plasmon resonance (SPR) peak centred at around 420 nm. The occurrence of peak at this wavelength (λmax value) reflects the size of AgNPs around 30–40 nm^20^. The influence of variation in concentrations of both CMT and silver nitrate was studied. The variation of concentration of CMT has not affected the AgNPs, however the variation of silver nitrate with respect to a fixed concentration CMT polysaccharide resulted in the gradual colour change to dark brown (Fig. 1a). This is due to the better seeding and higher yield of AgNPs (Fig. 1a) which is typically facilitated in presence of CMT polysaccharide. UV-visible spectra acquired 6 months after post-synthesis of these AgNPs suggest that these particles are stable at room temperature (Fig. 1b). The DLS analysis was carried out to assess the size and dispersity pattern of silver nanoparticles. The DLS result reveals particle sizes which are the sizes of the shell, while the real sizes of AgNP cores are smaller (Fig. 1c). Rise in CMT concentration, increases reactive –OH concentration in the medium which accelerates AgNP formation and subsequent inter-particle aggregation. Further, DLS measurements can indicate the hydrodynamic volume representing the size of overall solvent associated nanoparticle and thus can provide qualitative information about the nanoparticles. The average size measured from DLS was found to be 128 nm in terms of percent intensity distribution and 10 nm by volume distribution. The poly-dispersity index (PDI) of 0.208 indicates the monodispersed pattern of nanoparticles^21^. The Zeta potential analysis also suggest that these AgNPs are stable in nature (Fig. 1d).

### FE-SEM and TEM analysis of CMT-capped AgNPs

**Figure 2:**
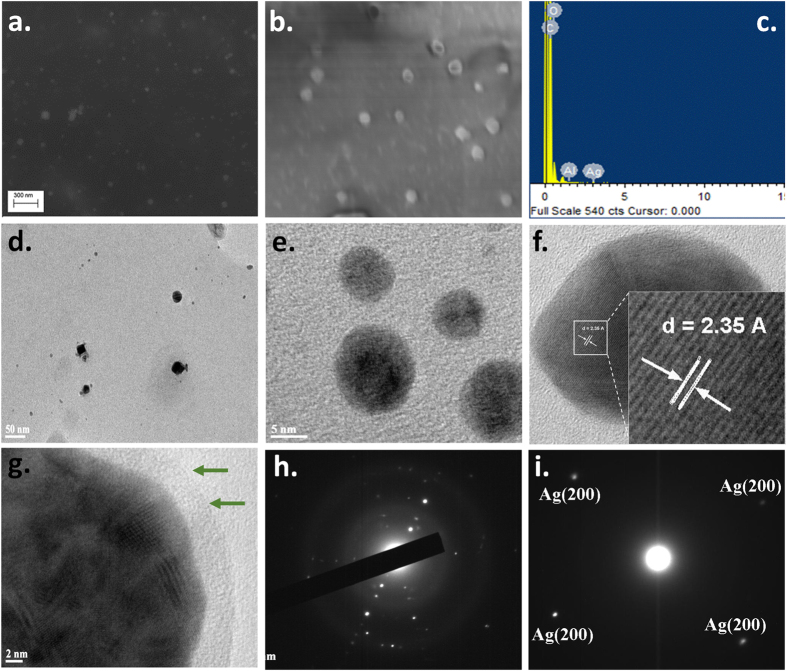
Electron microscopic characterization of CMT-capped AgNPs. (**a**,**b**) FE-SEM images of silver nanoparticles. (**c**) EDX pattern of silver nanoparticle. (**d**,**e**) TEM images of AgNPs in low and high magnification. (**f**,**g**) High resolution TEM image demonstrating the lattice pattern and the presence of CMT-cap (indicated by green arrows) on the AgNP. (**h**,**i**) SAED pattern of AgNP.

To confirm further the dispersion and sizes of these NPs, we performed FE-SEM and TEM. The FE-SEM image (Fig. 2a) shows that the nanoparticles are mostly spherical or polygonal in shape. This observation is further corroborated by TEM analysis. The TEM images show that the nanoparticles formed are of different sizes but mostly spherical and polygonal in shape (Fig. 2d–f). The selected area electron diffraction (SAED) shows specific spots corresponding to Ag interfacial layers in diffraction mode (Fig. 2h,i) and bright-field images (Fig. 2f) show multiple lattice domains, indicating polycrystalline nature of silver (Fig. 2). The average size of AgNP was found to be 30–40 nm. The high resolution lattice image confirms the presence of Ag(111) phases with a lattice constant of 0.235 nm. The EDX spectrum indicates the presence of silver nanoparticles in polymer capping (Fig. 2c). The relative abundance of elemental carbon and oxygen may be attributed to the presence of capping agent CMT polysaccharide which forms the shell surrounding the silver nanoparticles forming the silver polymer nanocomposites. The TEM images also confirm the physical presence of CMT capping on the AgNPs (Fig. 2g).

### Stability of CMT-capped AgNPs

The long-time stability of CMT-capped AgNP is established by surface plasmon resonance observed from UV-Vis spectral analysis. The SPR data collected after 6 months of synthesis of AgNPs was compared with that of AgNP synthesized initially. The SPR with λmax 418 remains nearly same indicative of unchanged λmax value, i.e. characteristic SPR of CMT-capped AgNPs (Fig. 1b). It suggests that the biopolymer CMT plays a key role in providing stability to AgNPs. The biopolymer forms ligand-shell surrounding AgNPs at the core forming silver polymer core-shell structure. The polymeric shell decreases surface potential that is responsible for accumulation of the silver nanoparticles to larger aggregates.

In order to understand the surface characteristics of the formed AgNPs and to correlate long- term stability, we performed Zeta Potential measurements. Dispersion with a low zeta potential value facilitates aggregation due to Vander Waal inter-particle attractions. The zeta potential value outside the range of −25mV to +25mV typically represents necessary electrical charge on the surface of nanoparticles required for higher degree of stability^22^. High value of zeta potential represents higher electrical charge on the surface of the nanoparticles, which causes strong repulsive forces among the particles that prevents agglomeration^23^. We observed a negative zeta potential value of about −36.7 mV that was observed for CMT-capped AgNPs (Fig. 1d). This value is well above the range of surface charge required for higher stability of nanoparticles. The higher degree of stability of these synthesized CMT-capped AgNPs correlates well with its higher surface charge as indicated by zeta potential value (Fig. 1).

### Antimicrobial efficacy of CMT-capped AgNPs

**Figure 3:**
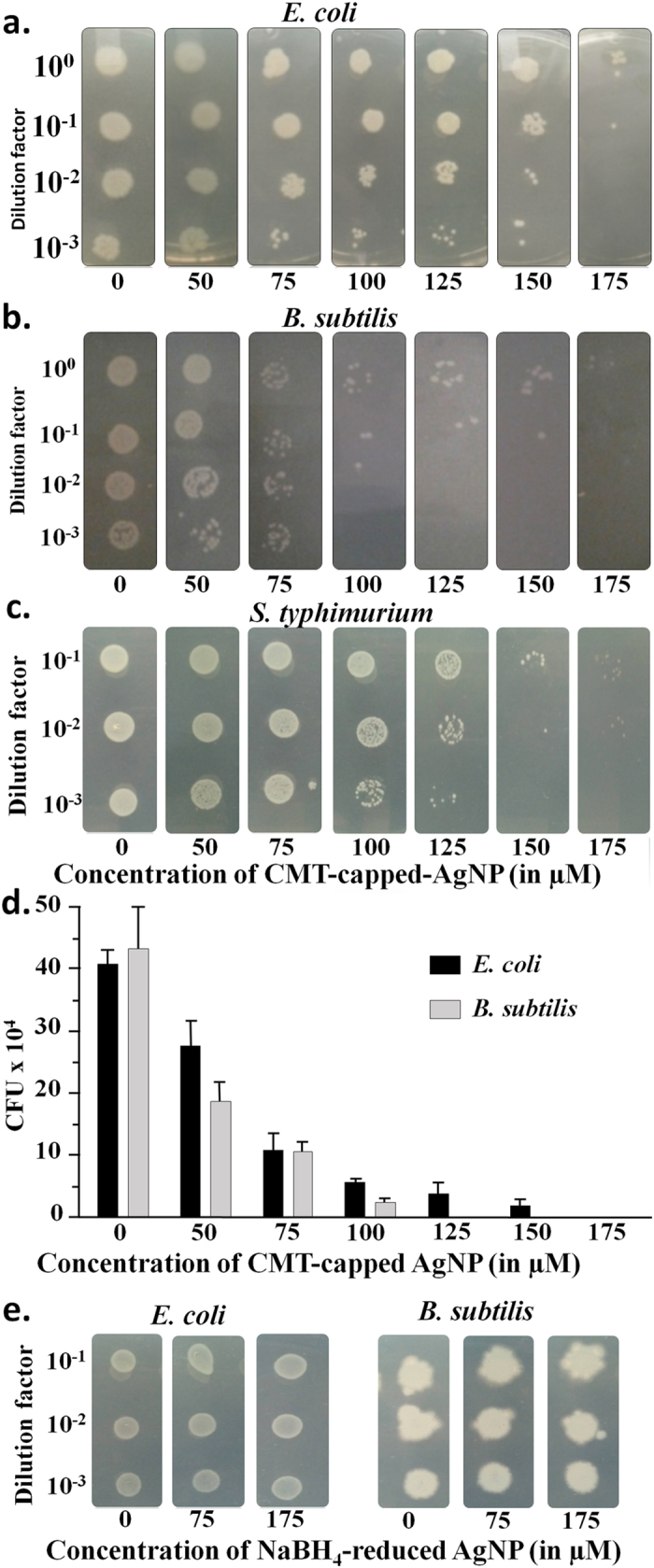
Antimicrobial efficacy of CMT-capped AgNPs. (**a**–**c**) CMT-capped AgNP concentration-dependent growth-inhibition of Gram negative *E. coli*, *Salmonella typhimurium* and Gram positive *B. subtilis* on LB agar plates. (**d**) CFU assay showing dose-dependent growth inhibition of *E. coli* and *B. subtilis*. (**e**) NABH_4_-reduced AgNPs are ineffective to inhibit the growth of *E. coli* and *B. subtilis* in the same concentrations.

The AgNPs were widely being known for their antimicrobial activity^7^,^24^,^25^. In this work we investigated the antimicrobial efficacy of CMT-capped AgNPs in the context of its dose-dependent effect on the growth of bacteria. The antibacterial activity of AgNPs were investigated against two bacterial strains, namely against *E. coli* and *B. subtilis* by colony forming unit assay (CFU). Bacteria at exponential phase were incubated for 3H with different concentrations of AgNPs. Subsequently the bacterial cells were grown in the presence of LB agar. The number of colonies formed on LB agar plates were analysed and surviving colonies were calculated after 12H of incubation. The AgNPs significantly inhibited *E. coli*, *B. subtilis*, and *Salmonella typhimurium* in a dose dependent manner (Fig. 3a–d). In case of 175 μM concentration there is no bacterial population observed even after 48 hours of culture (Fig. 3). However, AgNP prepared by NABH_4_–mediated reduction method neither inhibits *E. coli* nor *B. subtilis* in this concentrations (Fig. 3f).

### Anti-biofilm activity of CMT-capped AgNPs

**Figure 4:**
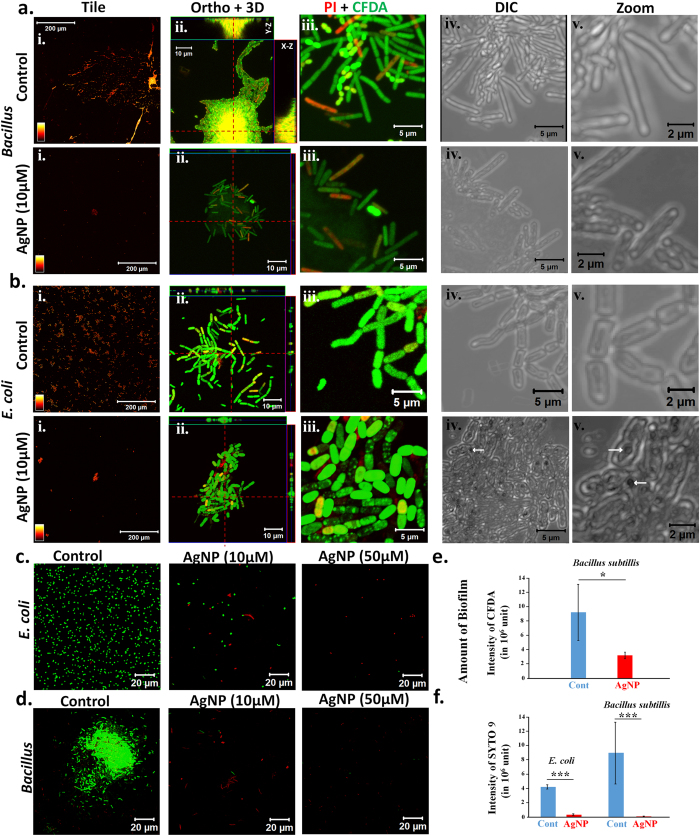
CMT-capped AgNP are effective against biofilm formation. (**a**,**b**) Shown are the confocal images of *Bacillus* (upper panel, (**a**)) or *E. coli* (lower panel, (**b**)) that were untreated or treated with sub-lethal concentration of CMT-capped AgNP (10 μM) for 3 hours and stained with CFDA (green, indicator of intact membrane, live cells) and PI (red, indicator of membrane damaged, dead cells). The left most panel (i) demonstrates the much larger view field and relative distribution of bacterial cells and biofilms. The 3D-confocal images with relative thickness (YZ and XZ images) of the biofilms are shown (ii). A larger view field and its corresponding DIC images are shown (iii-iv). The much enlarged DIC image shows the morphology of the individual bacterial cells (v). Arrows indicate the AgNPs that are present at the surface or within the bacterial cell. (**c**,**d**) Live-dead analysis of *E. coli* and *Bacillus* demonstrating the concentration dependent growth of these strains. (**e**) Quantification of biofilm formed by Bacillus in absence and presence of CMT-capped AgNP (n = 4, P value is <0.05). (**f**) Quantification of live *E. coli* and *Bacillus* cells in absence and presence of CMT-capped AgNP (n = 10. P values are <0.001).

Next we tested the effect of AgNPs on the biofilm formation by bacteria. As no bacterial colony was observed in AgNP at moderate or higher concentrations, for these experiments we used sub-lethal concentration of AgNP (10 μM) for very short time (3H) or 24H. For that purpose we have used two different species, namely *Bacillus subtilis* and *E. coli*. Cells were subjected to staining by propidium iodide (PI, stains membrane damaged, dead cells) and 5(6)-Carboxy fluorescein diacetate (CFDA, stains membrane intact, live cells) and fixed with 2% paraformaldehyde before processing for imaging. From the Z-stacked images obtained using confocal microscopy, we noted that *Bacillus* can form thick (as high as 10 μm) biofilm in control conditions and cells have relatively smaller size, clear septum and smooth surface morphology. In control conditions *E. coli* cells too show well dispersed cells with clear septum and smooth surface morphology. In contrast, in presence of sub-lethal concentration of AgNPs, biofilm formation is completely inhibited, and only very few *Bacillus* or *E. coli* cells were observed (Fig. 4). These cells reveal relatively elongated size, no clear septum and ruffled surface morphology (Fig. 4). These in general suggest that this CMT-capped AgNPs at a very low concentration can also prevent bacterial cell division, cause membrane damage and prevent their association to form biofilms. The quantification of biofilm formed by *Bacillus subtilis* also confirms that CMT-capped AgNPs are effective against biofilm formation (Fig. 4e). Similarly, the quantification of live cells using a more sensitive “live-dead analysis” also confirms that CMT-capped AgNP effectively inhibits the growth of *Bacillus* and *E. coli* in much lower concentrations (Fig. 4c,d,f).

### Effect of CMT-capped AgNPs on bacterial cell division and membrane damage

**Figure 5:**
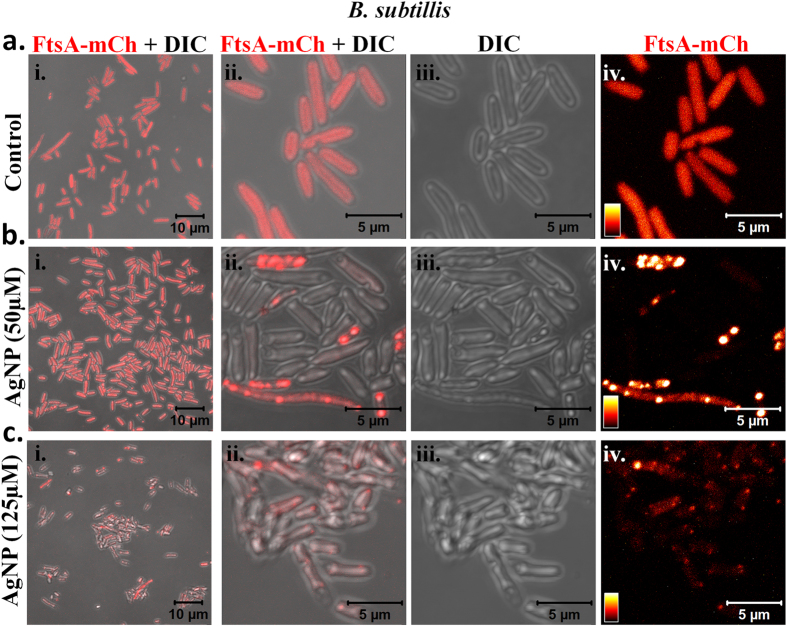
CMT-capped AgNP alters expression and localization of bacterial cytoskeletal protein FtsA. *E. coli* cells stably expressing FtsA-mCherry (from *Bacillus subtillis*) (Red) were grown in control conditions ((**a**), upper panel) or treated with 50 μM ((**b**), middle panel) or with 125 μM ((**c**), lower panel) AgNPs for 3H and analysed by confocal microscopy. DIC images merged with fluorescence images of respective view fields (left most) or enlarged view fields are shown. Much enlarged view fields of the corresponding samples and the respective fluorescence intensity (depicted in intensity scale) are shown in the right most side. Clustering of FtsA-mCherry in response to AgNP is visible as white spots.

**Figure 6:**
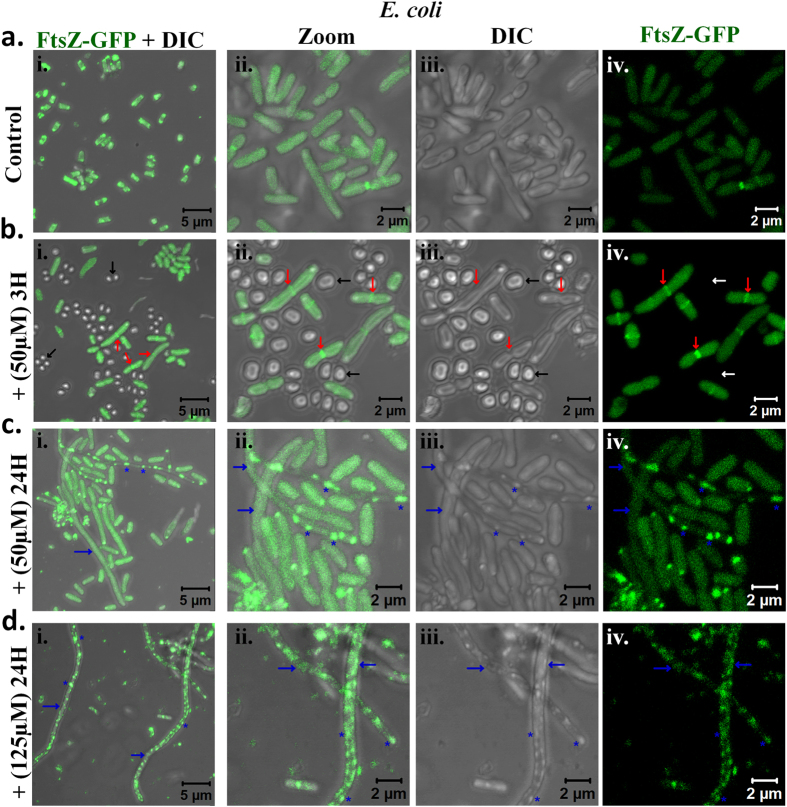
CMT-capped AgNPs alter expression and localization of bacterial cytoskeletal protein (FtsZ) responsible for cell-division. (**a**,**b**) *E. coli* cells stably expressing FtsZ-GFP (green) are grown in control conditions ((**a**), upper panel) or treated with 50μM AgNP for 3H (panel (**b**)) and analysed by confocal microscope. Several mini-cells without any FtsZ-GFP (indicated by black arrows) and few elongated cells with accumulated FtsZ-GFP at the septum in the middle region (indicated by red arrows) is developed within 3H of treatment. (**c**,**d**) Treating cells with CMT-capped AgNPs for 24H at moderate (**c**) or at higher dose (**d**), very few but elongated bacterial cells (indicated by blue arrows) with much clustered and distorted localization of FtsZ-GFP at the membranous region (indicated by asterisks *) is visible.

In order to probe if AgNP really affects the bacterial cell division machineries, we used *E. coli* which express FtsA-mCherry (from *Bacillus subtillis*) or FtsZ-GFP (from *E. coli*) after stable integration^26^,^27^. We noted that most cells have much higher expression and a uniform distribution of FtsA-mCherry in control conditions suggesting that the cells are in mostly log phase of their growth. However, treating cells with AgNPs even at sub-lethal concentration (50 μM), the FtsA-mCherry expression diminished and/or get clustered at corners or other points (Fig. 5). At non-permissible concentrations (125 μM), within 3H the cells reveal almost no expression of FtsA-mCherry and membrane damage becomes prominent. Few cells that are visible in this condition are much elongated and reveal no septum formation suggesting that the CMT-capped AgNP blocks bacterial cell division in general. In order to confirm that this CMT-capped AgNP is indeed blocking bacterial cell division, we explored the effect of the same on the expression and localization of FtsZ-GFP. We noted that in control conditions, most of the cells express FtsZ-GFP uniformly, have uniform size and shape, suggesting that most of the cells are indeed in log phase of their growth and cell division occurs routinely at a certain cell volume and surface ratio. However, treatment of the cells with 50 μM AgNPs for 3H only results in formation of much smaller sized cells that express no FtsZ-GFP. These cells are very similar to what has been proposed as “mini-cells”^28^. Few cells which express FtsZ-GFP in these conditions; become elongated and Z-ring is visible in these cells (Fig. 6). However, increasing the duration to 24H, both at lower concentration and intermediate concentration (yet below MIC value) results in formation of few but much elongated cells. Some of these elongated cells are even more than 50 μm in length. The localization of FtsZ-GFP is visibly not uniform in these cells and often condensed clusters or partly broken Z-ring-like structures in the certain positions are visible in these cells. The surface morphologies of these cells are not uniform and localization of FtsZ-GFP correlates well with the deformed bacterial surface as observed by high-resolution DIC images. These results confirmed that CMT-capped AgNPs reveal antibacterial properties by altering bacterial cell division machineries consisting FtsA and FtsZ.

**Figure 7:**
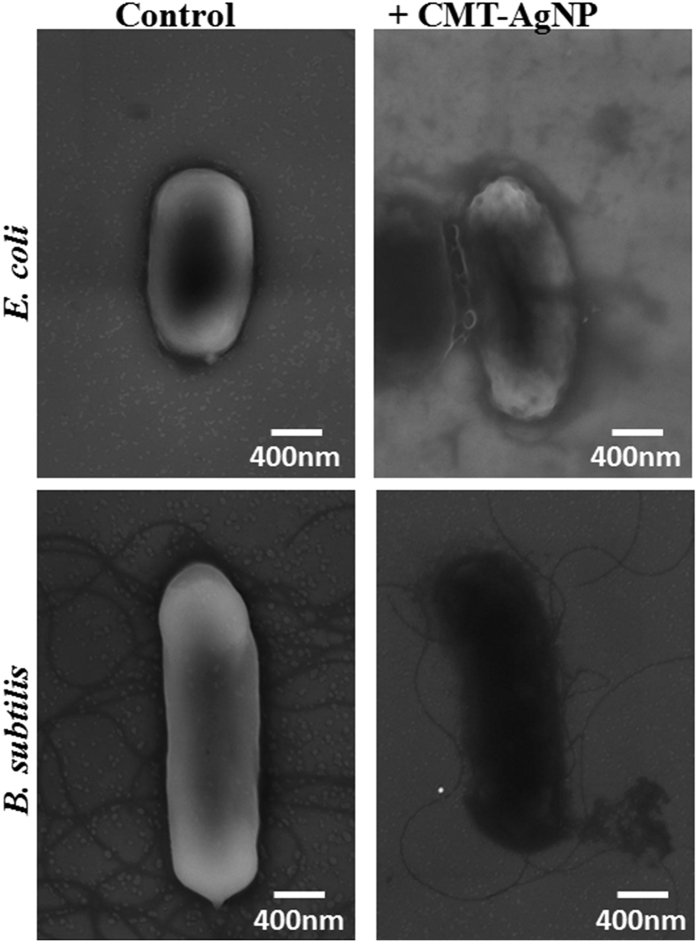
CMT-capped AgNP cause bacterial membrane damage. SEM analysis of *E. coli* and *Bacillus subtillis* cells treated with CMT-capped AgNP for 3 hours and processed for SEM analysis. The scale bars correspond to 400 nm.

In many cases, we observed enhanced permeability of PI in CMT-capped AgNP-treated bacterial cells. Also PI staining is observed in CFDA positive cells too, suggesting that CMT-capped AgNP may cause membrane damage. In order to explore that we analysed CMT-capped AgNP-treated *E. coli* and *Bacillus subtillis* cells by SEM. This analysis confirms that CMT-capped AgNP cause membrane damage (Fig. 7).

### Efficacy of CMT-capped AgNPs on multiple drug resistant bacterial strains

**Figure 8:**
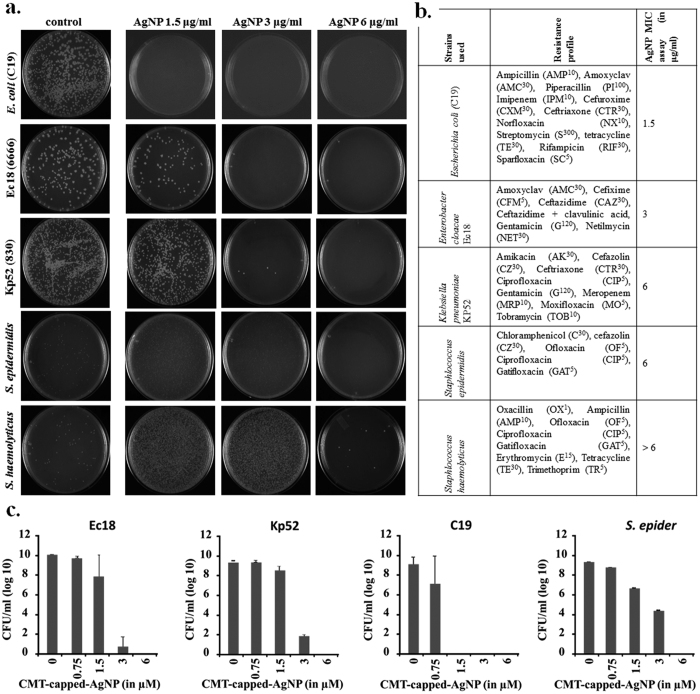
CMT-capped AgNP is effective against different MDR bacterial strains. (**a**) Shown are the growth pattern in plates in absence and presence of increasing concentrations of CMT-capped AgNP. (**b**) Detailed antibiotic-resistance profile and MIC values against CMT-capped AgNP of these strains are indicated. (**c**) CMT-capped AgNP inhibits multiple drug resistant bacterial strains in a dose-dependent manner. All values are statistically significant (n = 4).

As the CMT-capped AgNPs block bacterial cell division and also reveal efficacy against biofilm formation, we tested the effect of the same on multi-drug resistance strains. Therefore, we used five different bacterial strains (*Staphlococcus haemolyticus, S. epidermidis, Escherichia coli* C19, *Klebsiella pneumoniae Kp52, and Enterobacter cloacae Ec18*) representing different genera and different drug-resistant profiles and explored the efficacy of CMT-capped AgNPs (Fig. 8, also see supplementary information). In comparison to the commonly used antibiotics, the CMT-capped AgNPs effectively inhibits the growth of these organism at concentrations of ~1.5 μg/ml (in some cases in ~6 μg/ml), indicating its efficacy to be used at this concentration *in vitro*. In contrast, uncapped AgNPs, such as AgNP produced by NABH_4_ reduction method cannot prevent the growth of MDR strains even in higher concentrations (Supplementary Figure 3). This observation was also confirmed by carrying out dilution plating of the inoculated test organism from tubes containing different antibiotics (Supplementary information).

### Effect of CMT-capped AgNP on mammalian cells

**Figure 9:**
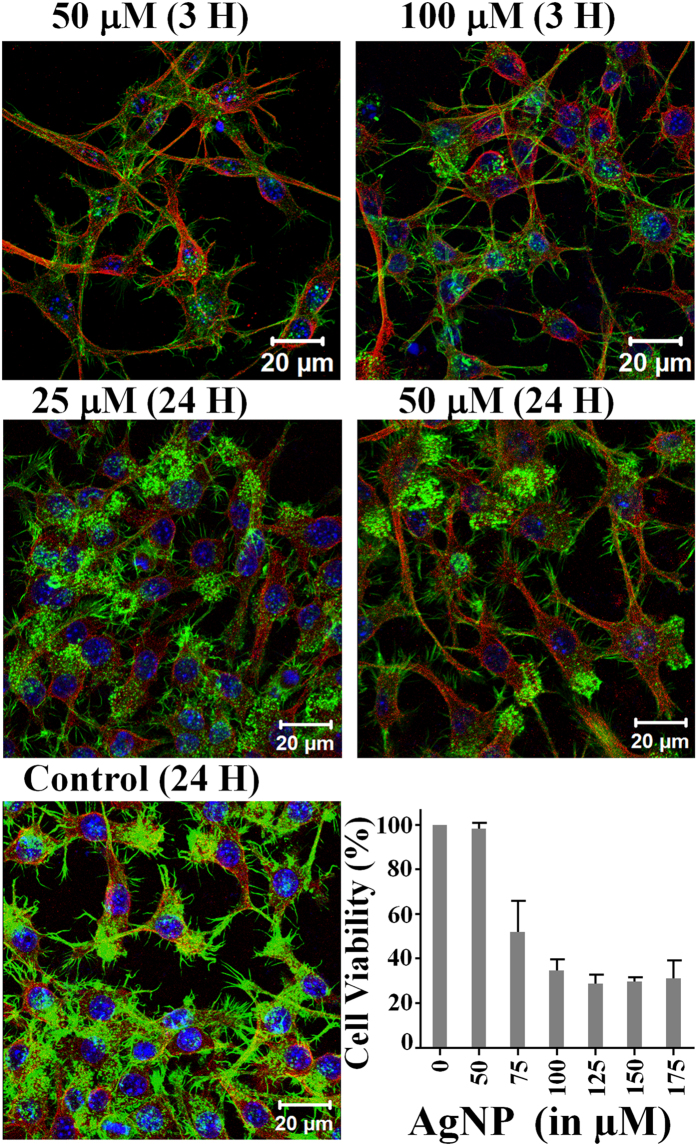
Bio-compatibility of CMT-capped AgNPs to mammalian cell. Shown are the confocal images of RAW 264.7 cells treated with CMT-capped AgNPs for different concentration and time duration followed by staining for actin cytoskeleton (green), microtubule (red) and DNA (blue). The percentage of cell viability assessed by MTT assay is presented as bar-graph at below (N = 4).

In order to explore the effect of this CMT-capped AgNP on eukaryotic cells, a number of mammalian cells were exposed to the AgNPs for different concentrations and durations. We analysed these cells by confocal microscope as well as by MTT assay (Fig. 9). Confocal imaging of actin as well as tubulin cytoskeleton suggest that CMT-capped AgNPs do not affect the mammalian cells significantly, especially when treated in low-dose yet higher than what is sufficient to achieve anti-microbial activities. Mouse macrophage RAW 264.7 cells remain unaffected when exposed for different concentrations and durations (for example 50 μM or 100 μM for 3H or for 25 μM or even 50 μM for 24H). Cell viability assays reveals that mouse macrophage RAW 264.7 cells are significantly tolerant to the exposure of higher concentrations of CMT-capped AgNPs. Neuronal cells (such as F11), Human keratinocytes (such as HaCaT cells) and Human osteoblasts (such as SaOS cells) also reveal that these cells are tolerant to this CMT-capped AgNPs to certain extent (data not shown). Notably, these concentrations are much higher than what is needed to prevent multiple antibiotic resistant bacteria suggesting that CMT-capped AgNPs may have several biomedical applications.

## Discussion

Though the commonly employed methods for the synthesis of metallic NPs are well established, these methods typically involve toxic and harsh chemicals, hazardous conditions, and costly apparatus. Chemical reduction of silver salts is one of the most commonly used method to prepare AgNPs. However, the specific uses of AgNPs are limited by their tendency to get aggregated to larger structures during post-synthesis phase. The unusual characteristics of nanoparticles and their respective applications are not feasible if these AgNPs are not protected from self-aggregation. So far various capping agents had been reportedly used such as surfactants, polymers, dendrimers, biological templates and biomacromolecules that protect these NPs from self-aggregation^29^. In comparison to the chemically synthesized AgNPs, the “green synthesis” of colloidal NPs involves biocompatible ingredients under physiological conditions of temperature and pressure. Moreover, the biologically active molecules involved in the green synthesis of NPs act as functionalizing ligands, making these NPs more suitable for biomedical applications, mostly due to their long-term stability. Therefore, the development of such protocols to synthesize nontoxic metallic NPs is currently of great interest and there is a huge demand for biosynthetic or green methods for such purposes.

Recently, many green synthesis processes have been reported as suitable alternative strategies for preparing AgNPs. For example, β-D-glucose has been used as a reducing agent for green synthesis of Ag nanoparticle where starch acts as a stabilizer^30^. Extract from acacia has also been used for green synthesis of AgNP where acacia extract acted as both reducing and stabilizing agent^31^. The “stabilization” and “controlled size” parameters of AgNPs are important prerequisites for specific end-use applications and such objectives can be achieved by choosing suitable reductant and capping agent in aqueous medium. So far, different polymeric stabilising agents, dendrimers, latex particles and microgels has been studied for controlling size parameter and to achieve better stabilization of silver nanoparticles^32^. Limited yet significant efforts have been channelized to explore new synthetic methodology involving *in-situ* reduction and capping of AgNPs, especially for *in situ* synthesis of AgNPs within the polymeric network architectures leading to new hybrids or composite systems^33^. In this context, the carriers, for example polymers, dendrimers or microgels, act as ‘nanoreactors’ that immobilize the particles and provide an easy handling.

So far different microorganisms (bacteria, fungi, virus), DNA and proteins have also been used for the synthesis of AgNPs and this is due to the strong affinity of Ag ions towards –SH, –NH_2_ and –COOH groups^34^. In these studies, the size of the NPs can be controlled by varying conditions such as pH, temperature, substrate concentration and reaction time. However, the microbial DNA and proteins exhibit immunogenic allergic reactions, thus restricting their usage for biomedical applications^35^. In this context, hydrogels are mostly safe, biologically inert and offer large free spaces between the cross-linked networks in the swollen stage that can act as “nanoreactors” for the nucleation and growth of the AgNPs^36^,^37^. However, this approach too has not been very successful due to the long reaction times, use of chemical and usually toxic reagents, low efficiency in converting the silver cations (Ag+) to nano-Ag, and lack of control over the size of AgNPs. Different biopolymers offers suitable environment for the synthesis of AgNPs and different nanocomposites due to their almost limitless availability, low price, and better biocompatibility. In this work we demonstrate that CMT polymer offers an ideal environment for synthesis of AgNPs that remain capped resulting in long-term stabilization.

As biomedical application of NPs are important aspects of recent nanotechnology approaches, development of next generation AgNPs and silver nanocomposites are mostly being attempted by using different biocompatible materials such as hydrogels. Literature suggests that various type of natural polymers like starch, poly ethylene glycol (PEG), chitosan and heparin can be used as both capping agent and reductant^38^,^39^. For example, Vimala *et al.* reported the preparation of semi-interpenetrating hydrogel networks through a redox polymerisation reaction using poly(acrylamide) and N,N-methylene bisacrylamide in the presence of different carbohydrate polymers such as gum acacia and carboxymethyl cellulose^35^. Similarly, other hydrogel silver nanocomposites have been synthesized by a synthetic route involving the formation of AgNPs within swollen poly(acrylamide-co-acrylic acid) hydrogels by using citrate ions^32^. These porous materials reduce silver cations inside the network using sodium borohydride. The amide and hydroxyl functional groups of the network were projected to enhance the stability of the AgNPs inside the matrix; however their size distribution was broad^32^. In this work, we have achieved AgNPs which are monodispersed, small in size and also with longer stability. The CMT polysaccharide used in this study efficiently reduced silver salt *in-situ* at moderate temperature without involving any external reducing agent. The carboxyl group present in CMT polysaccharide plays a key role by increasing the solubility of polysaccharides in aqueous media and also by increasing the stability of polysaccharide. It also helps in complex formation with the silver during the *in-situ* reduction process. This suggests that CMT is a viable option as a reductant for the green synthesis of AgNPs.

In general, the effectiveness of nanoparticles against microbes becomes limited when AgNPs synthesised within hydrogel networks (as nanoreactors) via *in situ* reduction of silver nitrate using different reducing agents^32^. This reduced activity of AgNPs has been attributed to the limited release of Ag+ ion from the AgNPs which are capped and protected by the hydrogel network. In contrast, in this work we demonstrate that CMT-capped AgNPs reveal excellent antimicrobial activity and is efficient against multiple drug resistant bacteria as well at low dose. This aspect of CMT-capped AgNPs also allowed us to explore the molecular mechanism by which AgNPs reveal antimicrobial activities. So far, there are three different possible molecular mechanisms proposed that can explain the antibacterial activities of AgNPs^16^ First mechanism suggests for a direct interaction of AgNPs with the bacterial cell membrane, which can cause subsequent membrane deformation, membrane damage and complex formation with components located inside the cells^39^. The second mechanism suggests for a possible interaction of AgNPs with thiol (−SH) groups present in proteins and production of reactive oxygen species (ROS)^40^. The third mechanism suggests the release of Ag ions which inhibit respiratory enzymes and also generate ROS^41^. The released Ag+ ion can also interact with thiol groups of proteins to inactivate vital proteins and to affect DNA replication^42^,^43^. However, so far very little is known about the exact molecular targets of the AgNPs in general.

The differential toxicity of AgNPs in general towards eukaryote and prokaryote can be attributed to presence of thiol groups in different extent in their membrane proteins^44^,^45^. It has been suggested that the exposed functional thiol groups present in prokaryote, form complexes easily with AgNPs which in turn cause death of prokaryotic cells^44^,^45^. Whereas absence of exposed functional thiol groups in membrane proteins causes lesser extent of damage to the eukaryotic cells.

**Figure 10:**
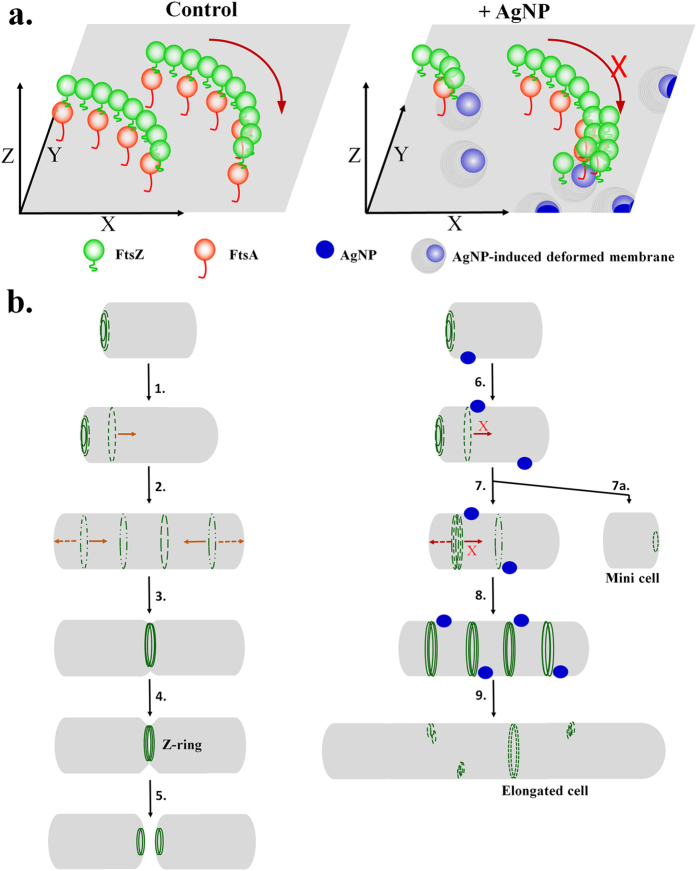
A schematic model demonstrating the plausible molecular mechanism of AgNP-mediated inhibition of bacterial growth. (**a**) FtsZ (green) & FtsA (red) form a membrane tethered molecular complex involved in the bacterial cell division. FtsZ-FtsA complex forms oligomeric structures which in turn forms ring-like structure and oscillate between two poles. Presence of AgNPs (blue) results in membrane deformation (grey rings with blue) and affects membrane tethering of FtsZ-FtsA complex as well as further movement of such oligomeric complexes. This results in inhibition of proper Z-ring formation and cell division. (**b**) Schematic representation of plausible mechanism of FtsZ-FtsA complex (green)-mediated bacterial cell division in absence (left side) and in presence of AgNP at sub-lethal concentration (right side). In absence of AgNP, oligomeric structures made of FtsZ-FtsA complex oscillate between two poles and form Z-ring structures at the centre resulting in proper and equal division when cells achieve a certain size. In presence of AgNP at sub-lethal concentrations, oscillation of the FtsZ-FtsA complex is inhibited and such complex become mislocalized leading to unequal cell division or no division resulting in the development of either much elongated cells or very small cells, i.e. mini-cells without any detectable FtsZ-FtsA complex.

Bacterial cell division is a tightly orchestrated process involving co-ordination of several cellular processes^46^. One of the key components of bacterial cell division are the proteins FtsZ and FtsA involved in bacterial cytokinesis^46^. Elongation of bacterial cell is often considered as a hallmark of altered morphology, where bacterial cell division is perturbed due to inhibition of FtsZ and FtsA protein functions^47^. Such elongated morphology may correlate well with the biofilm formation and/or with the resistance against antibiotics. Using CMT-capped AgNPs at sub-lethal concentration, our work suggest that FtsZ-FtsA complex is a major molecular target for AgNPs. Other studies have demonstrated that AgNPs are able to penetrate bacterial cell membranes to cause increase in permeability and disturbance in respiration, which lead to cell death. However, in this work our high-resolution DIC images suggest that CMT-capped AgNPs are most likely attached at the outer surface of the bacterial membrane and therefore are able to act even from outside. SEM images also suggest that CMT-capped AgNPs induce membrane damage. Based on the results and other literature, we propose a model suggesting how CMT-capped AgNP can affect the bacterial cell division (Fig. 10).

We propose that sub lethal exposure of CMT-capped AgNP alters the bacterial morphology and alters the expression as well as localization of FtsZ and FtsA, and thereby blocks bacterial cell division. FtsZ and FtsA represent essential and well conserved ancient bacterial cell division machineries which associates with bacterial membrane and forms Z-ring structure. Membrane association of CMT-capped AgNP (even at the outer surface) seems to affect proper oligomerization, oscillation of oligomeric structures as well as subsequent complex formation of FtsZ and FtsA leading to block in cell division^48^. In this context, it is important to mention that high resolution electron microscope images confirm that FtsA-FtsZ complex tethers to membrane and such membrane association is essential for proper force generation required for further membrane constriction and subsequent cell division^27^,^49^,^50^,^51^. Such membrane association is in general required for bacterial cell division which may be even independent of the size of the bacterial cells^52^. Involvement of other cell division regulatory proteins such as MinC, MreB etc. in the process of AgNP-mediated blockage of cell division cannot be ruled out^53^,^54^,^55^,^56^. However, involvement of bacterial membrane and membrane-bound proteins such as ZipA in the proper localization of FtsZ has been established^57^,^58^. Nevertheless, due to its involvement in bacterial cell division, FtsZ and its molecular complexes have been recently considered as an realistic molecular target for potential antibacterial drugs^59^,^60^. In this context, our results indicate that membrane damage and mislocalization of FtsZ can be a general mechanism by which CMT-capped AgNPs can execute the antibacterial activity. Nevertheless, in this work we describe the synthesis, characterization and antimicrobial application of a newly developed AgNP. We demonstrate that this CMT-capped AgNP can have potential usage as a suitable agent at very low dose against multiple drugs resistant bacteria. Such properties may have a wider application in diverse fields such as biomedical, textile and also in corrosion control.

## Materials and Methods

### Materials

Carboxy Methyl Tamarind (CMT) polysaccharide powder was procured as a gift item from Hindustan Gum & Chemical Pvt. Ltd. (Ahmadabad, India). Silver nitrate (AgNO_3_) was purchased from Sigma Aldrich (Bangalore, India). Milli-Q water is used for preparing all the solution. All other chemicals were purchased from SD. Fine Chemicals (Mumbai, India) if not mentioned otherwise. Propidium Iodide (PI) and CFDA dye was purchased from Sigma Aldrich (Bangalore, India).

### Synthesis of silver nanoparticles

CMT polysaccharide powder was dissolved in Milli-Q water to make a homogenous stock solution of different concentration (0.1–0.5%w/v) with constant stirring on magnetic stirrer at 60 °C for 1H. AgNO_3_ (50 mM) stock solution was also prepared using Milli-Q water. The synthesis of silver nanoparticle involves dissolution of a fixed concentration of silver nitrate (AgNO_3_) containing various concentration of CMT solution followed by autoclaving at 121 °C and 15psi. The same procedure was followed keeping CMT solution concentration fixed with varying concentrations of silver nitrate solution. These two different parameters namely concentration of silver nitrate (AgNO_3_) and concentration of CMT solution were optimised for desired result.

### Characterization of Silver Nanoparticles

Synthesis of nanoparticles was evaluated by ultraviolet-visible spectroscopy (Cary 100 Agilent technology, USA). The brown coloured solutions containing polymer-capped silver nanoparticles were centrifuged at 12000 RPM (Sigma 3218K) and the supernatants were taken for UV-visible Spectral analysis (300–700 nm). The surface plasmon resonance (SPR) was observed for polysaccharide-capped silver nanoparticles (AgNP) against the only CMT polysaccharide solution (used as blank). Further, average hydrodynamic volume (and hence the average size) and dispersity index of the nanoparticle were obtained by DLS Zetasizer (Zetasizer Ver. 7.03, Malvern instruments). The surface charge was measured from DLS Zetasizer. The elemental composition of CMT-capped silver polymer nanocomposite were analysed from EDX analysis (Zeiss SIGMA). The long-time stability of CMT-capped silver nanoparticles was evaluated by UV- visible spectral analysis.

### Microscopic characterization of AgNPs

The pattern of distribution within the polymer matrix and dispersity of AgNPs were analysed by field emission scanning electron microscopy (FE-SEM) (Zeiss SIGMA) and high resolution transmission electron microscopy (HRTEM) with 200 keV electrons (2010, JEOL URP pole piece to give point to point resolution of 0.19 nm).

### Measurement of cell viability by MTT assay

Cytotoxicity evaluation of polymer-capped AgNPs was examined by using MTT assay as per standard protocol^61^. The MTT salt [3-(4,5-dimethylthiazol-2-yl)-2,5-diphenyl tetrazolium bromide (M.P Biochemicals) is reduced by mitochondrial dehydrogenases to the water insoluble MTT formazan.Approximately10^4^cells (Mouse macrophage RAW 264.7) were seeded in flat-bottomed 96-well polystyrene coated plate (Tarsons Products Pvt. Ltd, Kolkata, India) and were grown for 24H at 37 °C in a 5% CO_2_ incubator. 1 mM stock solutions of nanoparticles were prepared in sterile distilled water. Series of dilutions equivalent to 50 μM, 75 μM, 100 μM or 125 μM of AgNPs in the medium was added to the plate and incubated for 24H again. Cells without nanoparticles were used as control. To determine the cell viability, MTT dye (100 μl from 0.1 mg/ml stock) was added to each well and incubated for 4H at 37 °C with 5% CO_2_ in dark. The formazan crystals developed as a result of cellular reduction of MTT were dissolved in dissolving buffer (4 g NP40 detergent in 50 ml 0.02 M HCl and 50 mL isopropanol) solution and incubated for 1H at 37 °C. The absorption was measured at 570 nm by using an ELISA reader (Biotek, Germany).The percentage of viable cells was calculated as cell viability (%) = Sample absorbance/control absorbance×100. The average values of 4 independent experiments (each time all conditions were repeated with triplicate wells) were considered.

### Antimicrobial efficacy

The antimicrobial efficacy of AgNP was evaluated by colony forming assay (CFU) study. The *E. coli* (1.5 ml) cultures were grown overnight and centrifuged at 5000 RPM for 5 minutes, washed with 1× PBS buffer, and again centrifuged at 5000 rpm for 5 minutes and the supernatant was discarded. Further added with 1× PBS (1 ml) buffer and the optical density was observed. Finally optical density (OD) of the sample was adjusted to 0.1 at 600 nm. 1 mM concentration of the AgNPs (equivalent to 50 μM, 75 μM, 100 μM, 125 μM, 150 μM, 175 μM respectively) and LB media with *E. coli* culture incubated for 3H. All samples were plated and incubated for 12H and number of colony forming units (CFUs) was calculated manually.

### Labelling of bacterial cells and imaging of biofilm

Bacteria which were either treated with 10 nM of AgNPs or left untreated and were incubated with CFDA (100 μM) and Propidium Iodide (5 μg/ml) in a shaking incubator at 37 °C for 60 min. Esterase activity of live bacteria cleaves CFDA to give green product that is retained in the bacterial membrane hence indicates viable bacteria^62^. Non-viable bacterial cells having compromised membrane facilitate entry of the membrane impermeable dye Propidium Iodide (PI).The cells were then fixed with 2% PFA for 20 min at room temperature and washed twice with PBS. Images were acquired using a Zeiss LSM780 confocal microscope using 63X objective. Images were processed in LSM software (Zeiss).

### MIC calculation against multi drug-resistant isolates

We evaluated the antimicrobial efficacy of AgNPs against bacterium C19 (identified as a multi-drug resistant *E. coli* strain, from laboratory culture collection of Dr. H. Mohapatra, NISER). The antibiogram profile of the organism as determined by disc diffusion method is shown in (Fig. 8b, also see Supplementary information). The *E. coli* strain MTCC 443 was used as a control. The bacterial cultures were revived in nutrient agar and nutrient broth till log phase. Following this, the cultures were sub-cultured in Muller Hinton Broth till OD_600nm_ = 0.6 and used for MIC determination by standard double broth dilution tube method^63^. MIC breakpoints for different antibiotic were determined following EUCAST, 2014, version 4.0 breakpoint. Disk diffusion has been done as described before and the interpretation has been done as per CLSI, 2014 guidelines^64^. The control strain used in the study is *E. coli* ATCC25922.

### Microbial culture, strains and imaging for bacterial cell division

LB media, agar-agar, yeast extract, trypton and NaCl, required for microbial culture were purchased from HiMedia Laboratories (Mumbai, India). Petri plates were obtained from Tarsons Products Pvt. Ltd (Kolkata, India). The *E. coli* strain expressing FtsZ-GFP (WM2724) was a kind gift from Dr. William Margolin (University of Texas Medical School, USA)^26^. The construct expressing mCherry-FtsA (pSZ50 BsFtsA) was a kind gift from Dr. Jan Löwe (MRC Laboratory of Molecular Biology, Cambridge, UK)^27^. BsFtsA construct was stably integrated into *E. coli* strain DH5α. Both *E. coli* strains were cultured in LB media and induced with 50 μM IPTG for 2H before adding AgNPs at the indicated concentrations (50 μM or 125 μM). The AgNP treated and untreated controls were incubated in shaking incubator (220 RPM, 37 °C) and live bacteria were immobilized on a pad of LB containing 1.5% low-melting-point agarose and covered with glass cover slips. The green and red fluorescence of the cytoskeletal proteins were detected using confocal microscope (LSM780, Carl-Zeiss, Germany) and the images were analyzed using LSM Image Browser software (Zeiss, Germany).

### Viability analysis of bacterial cells

The bacterial samples (with or without treated with CMT-AgNP for 3 hrs) were stained with LIVE/DEAD BacLight Bacterial Viability Kit (L7012; Molecular Probes, Invitrogen, Grand Island, NY, USA) as per the manufacturer’s protocol. This kit contains a green fluorescent dye SYTO 9 (which permeates both intact and damaged membranes of the cells, binds to nucleic acids and is a marker for live cells) and a red fluorescent dye propidium iodide (PI, which enters only non-viable cells with significant membrane damage). The stained cells were imaged using Zeiss LSM 780 Laser Scanning Confocal Microscope.

### SEM of bacterial cells

*E. coli* and *Bacillus subtillis* were either left untreated or treated with CMT-capped AgNP for 3 hrs and then collected by centrifugation, washed twice with 0.85% NaCl, and fixed with 2% glutaraldehyde at room temperature (~25 °C). The cells were resuspended in double distilled water and dehydrated on silicon substrate chips. Surface morphology, shape, and size of the bacterial cells were examined using field emission gun based scanning electron microscopy (FEG-SEM) equipped with BSE detector, with 5 kV electrons (Neon 40 cross-beam system, M/S Carl Zeiss GmbH).

## Additional Information

**How to cite this article**: Sanyasi, S. *et al.* Polysaccharide-capped silver Nanoparticles inhibit biofilm formation and eliminate multi-drug-resistant bacteria by disrupting bacterial cytoskeleton with reduced cytotoxicity towards mammalian cells. *Sci. Rep.* **6**, 24929; doi: 10.1038/srep24929 (2016).

## Supplementary Material

Supplementary Information
