# Supplementary material for: Polysaccharide-capped silver Nanoparticles inhibit biofilm formation and eliminate multi-drug-resistant bacteria by disrupting bacterial cytoskeleton with reduced cytotoxicity towards mammalian cells
Source: Sci Rep. 2016 Apr 29;6:24929. doi: 10.1038/srep24929 (PMC4850392; doi:10.1038/srep24929)
Supplement: Supplementary Information [file srep24929-s1.docx]

***Supplementary information:***

**Polysaccharide-capped silver Nanoparticles inhibit biofilm formation and eliminate multi-drug-resistant bacteria by disrupting bacterial cytoskeleton with reduced cytotoxicity towards mammalian cells**

**Sridhar Sanyasi^1*^, Rakesh Kumar Majhi^2*^, Satish Kumar^1^, Mitali Mishra^2^, Arnab Ghosh^3^, Mrutyunjay Suar^1^, Parlapalli Venkata Satyam^3^, Harapriya Mohapatra^2^, Chandan Goswami^2🖂^, Luna Goswami^1,🖂^**

1. School of Biotechnology, KIIT University, Patia, Bhubaneswar 751024, India

2. School of Biological Sciences, National Institute of Science Education and Research, Institute of Physics Campus, Sachivalaya Marg, Bhubaneswar 751005, India

3. Institute of Physics, Sachivalaya Marg, Bhubaneswar 751005, India

* Equal contribution

^🖂^Address for correspondence: [*chandan@niser.ac.in*](mailto:chandan@niser.ac.in)*,* [*lbh_1975@yahoo.co.in*](mailto:lbh_1975@yahoo.co.in)

**S Table 1. Shows optimization parameters of silver nanoparticle synthesis.**

| **CMT % (w/v)**  **(Fixed Concentration)** | **AgNO_3_**  **Concentration** | **CMT polysaccharide % Solution (w/v)** | **AgNO_3_**  **Concentration** | **Batch volume of reaction mixture (in ml)** |
| --- | --- | --- | --- | --- |
| **0.1%** | 1 mM | 0.1% | 1 mM | 20 |
| **0.1%** | 2 mM | 0.2% | 1 mM | 20 |
| **0.1%** | 3 mM | 0.3% | 1 mM | 20 |
| **0.1%** | 4 mM | 0.4% | 1 mM | 20 |
| **0.1%** | 5 mM | 0.5% | 1 mM | 20 |

**Supplementary Table 2: MIC assay for antibiotics for MDR strains**

**
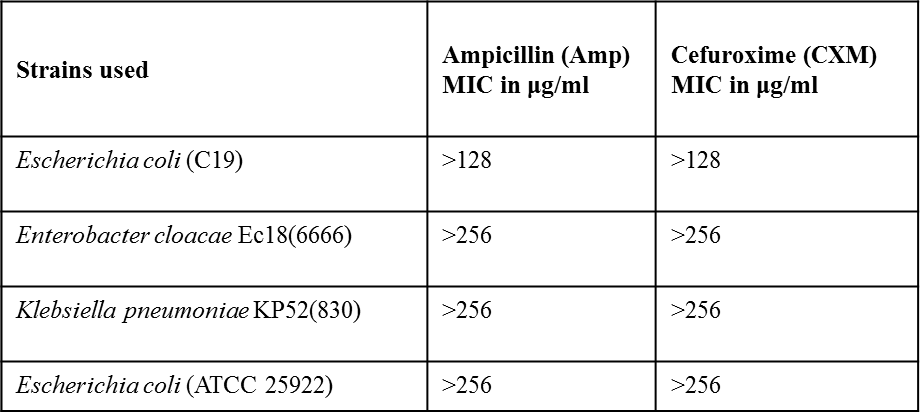
**

**Supplementary table no. 3: MIC assay for NaBH_4_-reduced AgNP against Kp52 (830)**

**
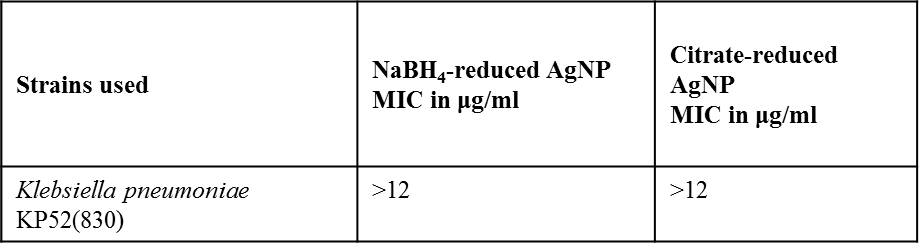
**

NaBH_4_-reduced AgNP was prepared as described in Mukherjee et al. (2014)


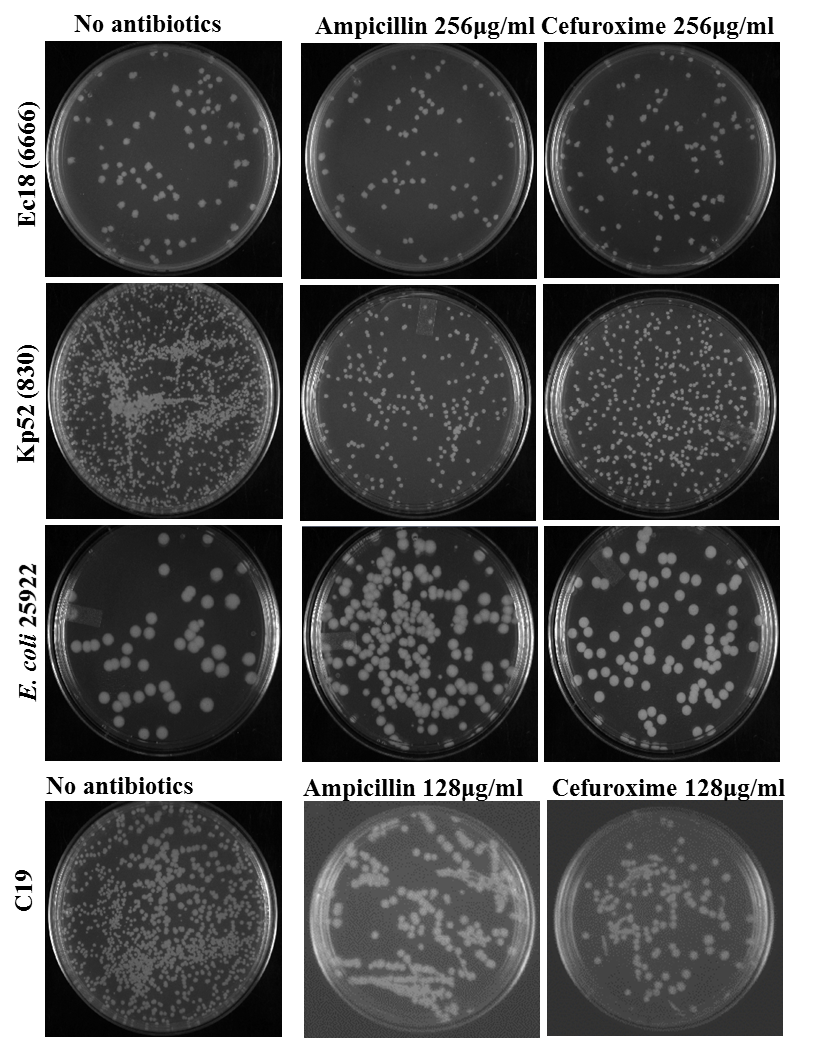


**Supplementary fig 1: Plate pictures of MIC assays with different MDR strains and control strain against different antibiotics.**

**
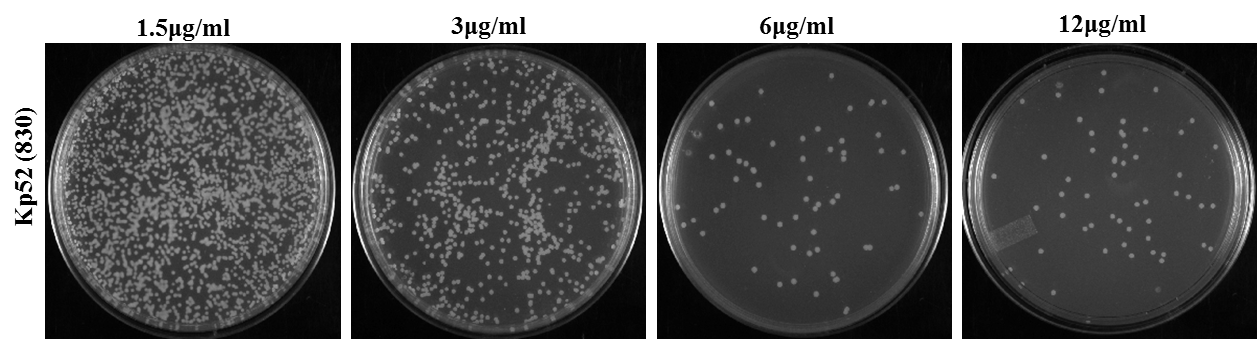
**

**Supplementary Fig 2: Plate pictures of MIC assay for NaBH_4_-reduced AgNP against Kp52 (830).** The NaBH_4_-reduced AgNP was prepared as described in Mukherjee et al. (2014)

**Supplementary information:**

Minimum Inhibitory Concentration (MIC) is defined as the lowest concentration of an antimicrobial drug that inhibits the visible growth of a microorganism following overnight incubation (Andrews JM, 2001). MIC is often used by diagnostic laboratories to confirm resistance. In addition, MIC is also used to assess the performance of a new antimicrobial agent and to determine the in vitro activity of the drug. Data obtained from MIC results are used to define breakpoint. Breakpoints are used to define susceptibility and resistance. According to the Clinical and Laboratory Standards Institute (CLSI), isolates that are not inhibited by usually achievable concentrations of the antimicrobial drug with normal dosage schedules are categorized as resistant (Turnidge and Paterson. 2007). Keeping in view the changing resistance patterns of organisms, breakpoints are revised taking data on each of the bacterial family. The EUCAST breakpoint table (EUCAST, 2014, version 4.0) has cited the breakpoint for Ampicillin and Cefuroxime to be 8mg/L for *Enterobacteriaceae*. Further, the zone diameter break point for 10ug and 30ug disc concentration of Ampicillin and Cefuroxime, has been set at 14- and 18mm respectively. Disk diffusion has been done as described before and the interpretation has been done as per CLSI, 2014 guidelines (Bauer et al. 1966, CLSI, 2014 Guide line). The control strain used in the study is *E. coli* ATCC25922.

**Refs:**

Andrews, J. M. (2001) Determination of Minimum Inhibitory Concentrations. *J. Antimicrob. Chemother.* ***48*** *Suppl 1*, 5–16.

Bauer AW, Kirby WM, Sherris JC, Turck M. (1966) Antibiotic susceptibility testing by a standardized single disk method. *Am J Clin Pathol*. **45**, 493-6.

CLSI, 2014 Guide line. (<http://clsi.org/wp-content/uploads/sites/14/2014/12/CLSI_Fall_2014_Catalog_Web1.pdf>)

Mukherjee S, Chowdhury D, Kotcherlakota R, Patra S, B V, Bhadra MP, Sreedhar B, Patra CR. (2014) Potential Theranostics Application of Bio-Synthesized Silver Nanoparticles (4-in-1 System). *Theranostics* **4**, 316-335.

Turnidge, J.; Paterson, D. L. (2007) Setting and Revising Antibacterial Susceptibility Breakpoints. *Clinical Microbiology Reviews* **20**, 391–408.
